# *Geste Antagoniste* Effects on Motor Performance in Dystonia—A Kinematic Study

**DOI:** 10.1002/mdc3.13505

**Published:** 2022-07-07

**Authors:** Rachel Newby, Siti Muhamed, Jane Alty, Jeremy Cosgrove, Stuart Jamieson, Stephen Smith, Peter Kempster

**Affiliations:** ^1^ Neurosciences Department Monash Medical Centre Clayton VIC Australia; ^2^ Department of Neurology Leeds Teaching Hospitals NHS Trust Leeds UK; ^3^ Hull York Medical School University of York York UK; ^4^ The University of Sheffield Sheffield UK; ^5^ Department of Electronic Engineering University of York York UK; ^6^ Politeknik Ungku Omar Ipoh Malaysia; ^7^ Wicking Dementia Research and Education Centre University of Tasmania Hobart TAS Australia; ^8^ Department of Medicine, School of Clinical Sciences Monash University Clayton VIC Australia

**Keywords:** dystonia, neurophysiology, movement disorders

## Abstract

**Background:**

The kinematic effects of *gestes* have not previously been studied. The mechanism(s) by which these sensory tricks modify dystonic movement is not well understood.

**Objectives:**

A kinematic investigation of the *geste* phenomenon in patients with dystonia.

**Methods:**

Twenty‐three patients with dystonia associated with a *geste* were studied. Twenty‐nine healthy controls also participated. Fifteen seconds of finger tapping was recorded by electromagnetic sensors, and the task was repeated with *geste*. Separable motor components were extracted using a custom‐written MATLAB script. Performance with and without *geste* was compared using Wilcoxon signed ranks testing.

**Results:**

Speed and fluency of finger tapping is impaired in dystonia. When patients executed their *geste*, speed of movement (amplitude × frequency) increased (*P* < 0.0001), and halts decreased (*P* = 0.007).

**Conclusions:**

That *gestes* improve not only dystonic muscle contraction but also the efficiency of voluntary movement suggests a broad influence at the premotor control stage.

Meige and Feindel coined the term *geste antagoniste efficace* to describe the “curious gestures” used by patients with torticollis to achieve transient relief from dystonic contraction.^1^ Alleviation of spasm could be observed even before the corrective maneuver was completed (before the hand touched the face). This, they argued, provided “conclusive evidence of the purely psychical value” of these acts. Kinnier Wilson, whose translation of Meige and Feindel's book and his subsequent writings crystallized *geste antagoniste* in neurological usage, recognized that it was not purely a motor phenomenon, and appeared to be driven by a range of adjustments in sensory feedback.^2^, ^3^

A *geste* is frequently observed in idiopathic focal dystonia, occurring in 70%–80% of those with cranial and cervical subtypes.^4^, ^5^ It is also well recognized in genetically‐based generalized dystonia.^6^ Many *gestes* produce a combination of tactile and proprioceptive sensory feedback from the body region chiefly affected by dystonia. *Geste*s may also involve visual, auditory or thermal stimuli. In the case of imaginary tricks, mental visualization of movement—without any change in sensorimotor feedback—is sufficient to alleviate dystonia.^7^

Previous kinematic research on ballistic and repetitive motor tasks show that there is slowness of voluntary movement in dystonia.^8^, ^9^, ^10^ No study has directly examined the influence of the *geste antagoniste* on motor performance.

## Methods

Thirty‐one patients with organic dystonia were recruited to a study of the kinematics of dystonia from movement disorders clinics at Monash Medical Centre in Melbourne Australia and Leeds Teaching Hospitals NHS Trust in the UK. Twenty‐nine healthy control subjects also participated. Diagnoses of dystonia had been made by movement disorders specialists, in line with consensus criteria.^11^ The presence of *geste antagoniste* in patients with dystonia was established by interview and observation. *Geste* was defined as “an episodic and specific maneuver that ameliorates dystonia in a manner not easily physiologically perceived as necessary to counteract the involuntary movement.”^7^ All participants gave written informed consent. Ethical approval was obtained from the Monash Health Human Research Ethics Committee (HREC code: 13424B) and the Yorkshire and Humber Sheffield Research Ethics Committee (HREC code: 14/YH/0143).

Participants held their arm with the elbow flexed and unsupported, palm facing the examiner and roughly in line with the shoulder. A Polhemus Patriot electromagnetic tracking sensor system (Polhemus Inc., Colchester, VT, USA) was connected to a tablet computer and placed on a table positioned between the participant's chair and the examiner. The sensors were secured over the dorsal aspect of the participant's thumb and index finger (over the nail bed) using Velcro straps. All tasks were undertaken with the dominant hand first, then repeated with the non‐dominant hand. The participant was asked to “tap your index finger and thumb as big and as fast as possible for 15 seconds, when I say begin”. This task was performed two more times. Approximately 20 min later, after completing a number of other movement tasks, subjects were asked to repeat the finger tapping whilst executing their *geste*. *Gestes* involving upper limb movement were performed with the opposite arm (i.e. the hand not connected to the sensors).

All kinematic measurement sequences were recorded on video, along with the videotape examination protocol that accompanies the Fahn‐Marsden Dystonia Rating Scale (FMDRS).^12^ Each video was assessed by two movement disorders specialists, who were blinded to the diagnosis. Scoring according to the FMDRS and the Movement Disorders Society Unified Parkinson's Disease Rating Scale (MDS‐UPDRS)^13^ item 3.4 (finger tapping) was carried out.

Kinematic recordings were transferred to a tablet computer for offline analysis. Pre‐processing was done to remove high‐frequency noise using a low‐pass (5 Hz) Butterworth filter. A custom MATLAB script determined tapping cycles as the period between two minimal separation points, indicating sequential index finger‐thumb oppositions. The following separable motor components were then extracted: overall speed (amplitude × frequency), rhythmicity (coefficient of variation for amplitude and velocity, by separating tapping cycles and dividing the standard deviation of maximum values by the mean of maximum values), halting tendency (percentage time spent at <5% of maximum velocity), and hesitations. The MATLAB code identified smaller peaks within each tapping cycle, which were counted as hesitations and totaled for all cycles.

The data was normalized to account for variability in hand size using the following formula:

$$\text{Normalized amplitude}=\left(D–D_{\min}\right)/\left(D_{\max}–D_{\min}\right).$$

where *D* = calculated separation distance, *D*_min_ = minimum separation distance and *D*_max_ = maximum separation distance. Normalized amplitude values range from zero to one and represents the distance between finger and thumb, relative to the anatomical dimensions of a participant's hand.

A related samples Wilcoxon signed ranks test was used to analyze the effect of *geste* in the dystonia group. For comparisons between patients and control subjects, Mann–Whitney testing was employed. To establish whether there was any significant variation across baseline trials (without *geste*), and between dominant and non‐dominant hands, a repeated measures ANOVA was applied, using HAND (dominant vs. non‐dominant) and TRIAL (1st, 2nd or 3rd) as within‐subjects factors. Although not all separable motor component measures were normally distributed, this approach was chosen as ANOVA is robust enough to allow for some deviations from normality, and relatively small sample sizes did not favor the use of an equivalent non‐parametric method. No significant difference in performance was noted across trials or between hands. The data was collapsed by intra‐subject averaging, across trials and hands in each individual for tapping without *geste*, and across hands for the *geste* task.

## Results

Twenty‐four patients with dystonia (15 female and 9 male, mean age 56) had a *geste*. The prevalence of *geste antagoniste* was therefore 77% in this study. One woman was unable to complete the task without using her *geste*, so her data was excluded from further analysis. Clinical details of the remaining 23 patients are shown in Table 1. Sixteen (70%) had upper limb dystonic activity, which was bilateral in nine.

**TABLE 1:** Clinical information, including *geste* characteristics

Clinical characteristics (including upper limb involvement)	Etiology	Age, gender	*Geste antagoniste*	FMDRS	MDS‐UPDRS 3.4
		Duration of dystonia (years)			Median (IQR)
Generalized *R,L*	Acquired (perinatal hypoxic brain injury)	52, M 52	Holding wrist	21	1.5 (1.00)
Segmental (cervical dystonia with dystonic upper limb tremor) *R,L*	Idiopathic	63, F 38	Touching chin	1.25	0 (0.75)
Focal (cervical dystonia)	Idiopathic	45, F 15	Touching chin	6.5	0 (0.00)
Focal (musician's hand dystonia) *R*	Idiopathic	36, M 0.4	Wearing splint/ holding forearm	1.75	0 (0.00)
Generalized *R,L*	Genetic (AD inheritance, mutation unknown)	42, F 31	Holding wrist	18.5	2.5 (1.75)
Right hemidystonia *R*	Acquired (left basal ganglia infarct)	39, F 16	Supporting arm (e.g. on pillow)	10.5	0.5 (1.00)
Segmental (cranio‐cervico‐brachial dystonia) *R,L*	Idiopathic	64, F 39	Holding forearm	10.5	1.5 (1.00)
Segmental (cranio‐cervical dystonia)	Genetic (AD inheritance, mutation unknown)	75, M 65	Holding chin	4.5	0 (0.00)
Segmental (cranio‐cervico‐brachial dystonia) *R*	Genetic (AD inheritance, mutation unknown)	67, F 54	Holding forearm	25	0.5 (1.75)
Focal (cervical dystonia)	Idiopathic	38, F 2	Holding chin	4	0 (0.00)
Focal (cervical dystonia)	Idiopathic	66, F 20	Holding back of head	8.75	0.5 (1.00)
Focal (cervical dystonia)	Idiopathic	76, F 23	Touching chin	8.5	0 (0.75)
Right hemidystonia *R*	Acquired (infantile traumatic brain injury)	74, F 73	Holding wrist	19.75	2 (4.00)
Focal (cervical dystonia)	Idiopathic	58, M 7	Touching cheek	9.75	0 (0.00)
Focal (writer's dystonia) *R*	Idiopathic	76, M 20	Holding wrist	0	0 (0.75)
Segmental (cervical dystonia with dystonic upper limb tremor) *R,L*	Idiopathic	62, F 35	Touching cheek	25.75	2 (1.50)
Focal (cervical dystonia)	Idiopathic	48, M 7	Touching cheek	11	0 (0.75)
Focal (musician's hand dystonia *R,L*	Idiopathic	35, F 21	Massaging arm/ pressure to certain points	2	0 (0.00)
Generalized dystonia *R,L*	Genetic (ADCY5 mutation)	29, M 29	Sitting up very straight	11.5	1 (0.75)
Segmental (cervical dystonia with dystonic upper limb tremor) *L*	Idiopathic	53, F 20	Holding neck	3.25	0 (0.00)
Segmental (writer's dystonia plus cervical dystonia) *R,L*	Idiopathic	70, M 30	Touching hand	12.5	0 (0.00)
Segmental (cervical dystonia plus writer's dystonia) *L*	Idiopathic	35, F 4	Touching chin	1	0 (0.00)
Segmental (cranio‐cervico‐brachial dystonia) *R,L*	Genetic (ANO3 mutation)	75, M 38	Resting head in hand	16.5	2 (0.75)

Distribution of upper limb dystonia, where present, is shown.

Abbreviations: M, male; F, female; IQR, interquartile range; R, right; L, left.

While all but one *geste* employed upper limb movement, in only 10 cases was the action directed towards an upper limb. Twelve *gestes* were directed at the cranio‐cervical region. Subjects who possessed a *geste* demonstrated, in comparison with those who did not, no significant differences in FMDRS or MDS‐UPDRS finger‐tapping scores.

Finger tapping without *geste* in dystonia subjects showed significant kinematic differences in comparison with controls. The dystonic group were slower (median speed 1.91 vs. 2.20; *U* = 174, *z* = −2.94, *P* = 0.003, *r* = −0.41) and more halting (median halts 5.45 vs. 4.96; *U* = 201, *z* = −2.44, *P* = 0.01, *r* = −0.34). MDS‐UPDRS finger tapping scoring, which had been performed without *geste*, also showed a small but significant increase in the dystonic group. Median score for each hand was 0.0 (interquartile range 1.5) in dystonia patients and 0.0 (interquartile range 0.0) in healthy controls (*U* = 196.5, *z* = −3.537, *P* < 0.001, *r* = −0.49).

**FIG. 1:**
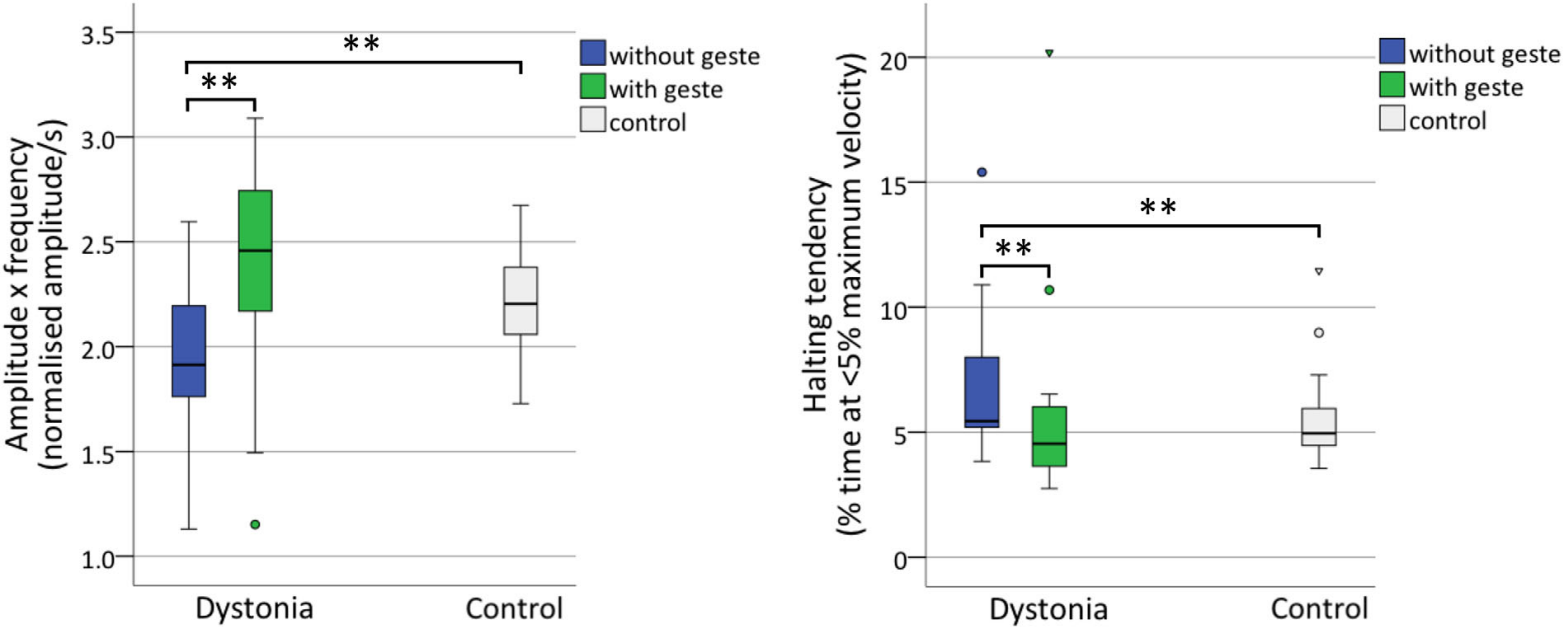
Boxplots for overall speed (amplitude × frequency) and halting tendency. Brackets and asterisks indicate statistically significant comparisons. Box = interquartile range (IQR); whiskers = highest and lowest values within 1.5 × IQR; band inside box = median. Outliers are denoted by triangles (>3 times IQR) and circles (1.5–3 times IQR).

In the dystonia group there was a significant effect of *geste*, with faster overall speed with *geste* (median 2.46) than without (median 1.91) (*z* = 4.02, *P* < 0.0001, *r* = 0.59) (see Fig. 1). There was no significant difference in speed between those with and without upper limb dystonia (*U* = 48, *z* = −1.05, *P* = 0.31, *r* = −0.02). Patients with dystonia also displayed reduced halting tendency when they activated their *geste*, median 4.54 versus 5.45 (*z* = −2.65, *P* = 0.007, *r* = −0.39).

As can be seen from Figure 1, performance with *geste* in dystonia subjects approximated levels achieved by healthy controls for both speed and fluency (halting tendency) of movement. A post‐hoc comparison of control against dystonia with *geste* revealed no significant difference for speed (*U* = 231, *z* = −1.89, *P* = 0.06, *r* = −0.26) or halting tendency (*U* = 268, *z* = −1.207, *P* = 0.23, *r* = −0.17).

There was no significant effect of *geste* on rhythmicity (coefficient of variation for velocity, *z* = −0.67, *P* = 0.52, *r* = −0.10; coefficient of variation for amplitude, *z* = −1.13, *P* = 0.27, *r* = −0.17) or number of hesitations (*z* = −1.71, *P* = 0.09, *r* = −0.25).

The analysis for variation across baseline trials (without *geste*), which is described in Methods, did not show evidence for a significant motor learning effect on separable motor component measurements (see Table S1).

## Discussion

Participants in this study fulfilled two criteria—a diagnosis of dystonia that complied with the 2013 Consensus Update^11^; and the presence of a *geste* according to an accepted definition of the phenomenon. As Table 1 shows, our dystonia group is composed of a range of disorders, in terms of both clinical characteristics and etiology. Task‐related and non‐task‐related dystonia are represented. Previous publications on sensory tricks emphasize their heterogeneity—in character of maneuver, topography, and type of dystonia.^7^ *Gestes* also occur in acquired dystonia, though reportedly at lower frequency than idiopathic forms.^14^ Three of our patients had a *geste* associated with acquired dystonia.

The mechanism(s) by which *gestes* alleviate dystonia is not known. There is neurophysiological evidence that intracortical facilitation is increased in dystonia, and that the execution of a *geste* restores a balance between facilitation and inhibition.^7^ This could involve normalization of altered gating of sensory input to motor circuits.^15^ These effects may be operating upstream of cortical motor output, consistent with the ability of multisensory or even imaginary tricks to reduce dystonic muscle contraction. An alternative hypothesis, based on analogy with the ocular motor system, conceptualizes cervical dystonia as the product of an unstable or “leaky” neural integrator in the brainstem, with feedback from a *geste* having a rectifying effect.^16^

It has been demonstrated that rapid voluntary movements are slowed in patients with dystonia.^8^, ^9^, ^10^ Our findings extend those observations in two ways. We did not recruit selectively for upper limb dystonia, unlike the previous studies. Yet we found alterations of speed and fluency of finger tapping, suggesting that dystonia's influences on movement are wide‐ranging. Dystonia does show other remote motor relationships, as, for instance, when a patient with cervical dystonia has tremor in a different body region.^17^ Secondly, we show that these effects are modified by enactment of a *geste*, which has not been reported before. Finger tapping improves with *geste* regardless of dystonia type or lateralization of upper limb dystonia. This improvement in hand kinematic function occurred even though the majority of maneuvers were directed towards body parts at some distance from the hand. In our dystonia group, effects on speed and fluency of hand tapping were substantial and statistically significant, reversing much of the “bradykinesia” of dystonia. That *gestes* improve not only dystonic muscle contraction but also the efficiency of voluntary movement gives support to a broad influence at the premotor control stage. It might be speculated that *gestes* correct basal ganglia sensori‐motor information flow in some general way, perhaps through effects on coding or compressibility of neural signaling.

### Study Limitations

There are pathophysiological differences across subtypes of dystonia,^18^ and *gestes* may operate differently in different subtypes. This study of a relatively small and heterogenous sample of patients with dystonia was not powered to detect such variations, should they exist. Since all subjects were tested without *geste* then with *geste*, it is not possible completely to exclude a motor learning effect as the basis for the improvement in motor performance. However, comparisons across sequential “freestyle” finger tapping tasks without *geste* showed no such effect. Since the finger tapping with *geste* was performed after a delay of approximately 20 min (during which subjects performed other experimental activities such as hand opening‐closing and pronation‐supination), it seems unlikely that motor learning could, on its own, account for this finding. Three subjects had cervical dystonia plus hand tremor associated with dystonia. Tremulous movements could have affected results, particularly measurements of rhythmicity and hesitation. It is generally agreed, though, that tremor can be a basic element or feature of a dystonic syndrome.^17^ Assessments were usually separated by at least 12 weeks from most recent botulinum toxin injections. While six subjects were studied within that interval, only two had received botulinum toxin injections to the upper limb.

## Author Roles

(1) Research Project: A. Study design, B. Data collection, C. Kinematic data processing, D. Clinical scoring; (2) Statistical Analysis: A. Design, B. Execution, C. Review and Critique; (3) Manuscript: A. Writing of the first draft, B. Review and Critique.

RN: 1A, 1B, 2A, 2B, 2C, 3A, 3B.

SM: 1C, 3B.

JA: 1A, 1D, 2C, 3A, 3B.

JC: 1D, 3B.

SJ: 2C, 3A, 3B.

SS: 1C, 2C, 3A, 3B.

PK: 1A, 1D, 2C, 3A, 3B.

## Disclosures

### Ethical Compliance Statement

Ethical approval was obtained from the Monash Health Human Research Ethics Committee (HREC code: 13424B) and the Yorkshire and Humber Sheffield Research Ethics Committee (HREC code: 14/YH/0143). All participants gave written informed consent. We confirm that we have read the Journal's position on issues involved in ethical publication and affirm that this work is consistent with those guidelines.

### Funding Sources and Conflicts of Interest

Dr Newby's salary was funded by the Monash Institute of Neurological Diseases. No conflicts of interest to disclose.

### Financial Disclosures for the Previous 12 Months

Stock Ownership in medically‐related fields: JA, SJ and SS: Shareholders in ClearSky Medical Diagnostics Ltd. Advisory Boards: JA: Abbvie, Merz. Employment: RN: Sheffield Teaching Hospitals NHS Foundation Trust; SM: Politeknik Ungku Omar, Malaysia; JA: University of Tasmania; JC: Leeds Teaching Hospitals NHS Trust; SJ: retired; SS: University of York; PK: Monash Health. Honoraria: JA: Allergan; JC: GE Healthcare. Royalties: JA: Taylor and Francis Publishing. Grants: JA: National Health & Medical Research Council, National Institute for Health and Care Research, and Royal Hobart Hospital Research Foundation.

## Supporting information


**Table S1.** Statistical analysis by Repeated Measures ANOVA for TRIAL (without *geste*) and HAND
