# Supplementary material for: Geste Antagoniste Effects on Motor Performance in Dystonia—A Kinematic Study
Source: Mov Disord Clin Pract. 2022 Jul 7;9(6):759–64. doi: 10.1002/mdc3.13505 (PMC9346233; doi:10.1002/mdc3.13505)
Supplement: Supplementary file 1 — Table S1. Statistical analysis by Repeated Measures ANOVA for TRIAL (without geste) and HAND [file MDC3-9-759-s001.docx]

**Statistical analysis by Repeated Measures ANOVA for TRIAL (without *geste*) and HAND.**

| Measure |  | |
| --- | --- | --- |
|  | TRIAL | HAND |
| amp x freq | *V* = 0.22 , *F*(2, 21) = 2.88, *p* = 0.08 | *V* = 0.29 , *F*(1, 22) = 0.67, *p* = 0.42 |
| COVamp | *V* = 0.06 , *F*(2, 21) = 0.67, *p* = 0.52 | *V* = 0.005 , *F*(1, 22) = 0.12, *p* =0.73 |
| COVvel | *V* = 0.03 , *F*(2, 21) = 0.32, *p* = 0.73 | *V* = 0.04 , *F*(1, 22) = 1.01, *p* = 0.33 |
| Hesitations | *V* = 0.10 , *F*(2, 21) = 1.10, *p* = 0.35 | *V* = 0.002 , *F*(1, 22) = 0.04, *p* = 0.84 |
| Halts | *V* = 0.06 , *F*(2, 21) = 0.64, *p* = 0.54 | *V* = 0.10 , *F*(1, 22) = 2.54, *p* = 0.13 |

amp = amplitude, freq = frequency, COVamp = coefficient of variation for amplitude, COVvel = coefficient of variation for velocity. V = Pillai’s trace test statistic.
